# Scientific and Ethical Issues in Mitochondrial Donation

**DOI:** 10.1080/20502877.2018.1440725

**Published:** 2018-03-12

**Authors:** Lyndsey Craven, Julie Murphy, Doug M. Turnbull, Robert W. Taylor, Grainne S. Gorman, Robert McFarland

**Affiliations:** Wellcome Centre for Mitochondrial Research, Institute of Neuroscience, Newcastle University, Newcastle upon Tyne, UK

**Keywords:** mitochondrial donation, ethics, human embryo, oocytes, maternal spindle transfer, pronuclear transfer

## Abstract

The development of any novel reproductive technology involving manipulation of human embryos is almost inevitably going to be controversial and evoke sincerely held, but diametrically opposing views. The plethora of scientific, ethical and legal issues that surround the clinical use of such techniques fuels this divergence of opinion. During the policy change that was required to allow the use of mitochondrial donation in the UK, many of these issues were intensely scrutinised by a variety of people and in multiple contexts. This extensive process resulted in the publication of several reports that informed the recommendations made to government. We have been intrinsically involved in the development of mitochondrial donation, from refining the basic technique for use in human embryos through to clinical service delivery, and have taken the opportunity in this article to offer our own perspective on the issues it raises.

## 1. Introduction

Mitochondrial disease is a term that encompasses a diverse group of genetic disorders characterised by mitochondrial dysfunction. These disorders show vast clinical heterogeneity, with patients presenting at any age and with a wide range of clinical features often affecting multiple organ systems (Gorman *et al.*^2016^). The symptoms are usually progressive and can be associated with severe morbidity and early mortality. Inherited mitochondrial disease is genetically heterogeneous and can be caused by a number of different genetic mutations found in either the nuclear DNA, which is inherited from both parents, or the mitochondrial DNA (mtDNA), which is inherited from the mother only. This has made the clinical diagnosis of mitochondrial disease challenging. Recent advances in next-generation sequencing technology have significantly improved the diagnostic rate for both patients and their families (Craven *et al.*2017a) but unfortunately, progress in this area has not been matched with the development of effective treatments. There is currently no cure for mitochondrial disease and clinical management is focussed on symptom alleviation. This highlights the importance of obtaining a genetic diagnosis as it opens up the possibility of genetic counselling to understand the risks of transmitting mitochondrial disease and allows reproductive options to be considered that will reduce this risk.

**FIGURE 1.**
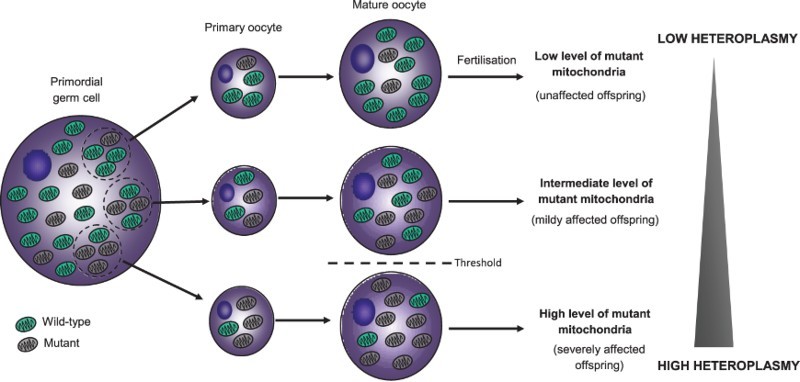
The mitochondrial genetic bottleneck, heteroplasmy and the threshold effect. Note: In patients who carry a pathogenic mtDNA mutation, the mutation can be present in all copies of the mitochondrial genome (homoplasmy) or only a proportion of the genomes (heteroplasmy). When heteroplasmy exists, it is the ratio of wild-type to mutant mtDNA that is important and clinical features will only manifest once a critical threshold level has been exceeded. The presence of a genetic bottleneck during early development means that women who carry a pathogenic mtDNA mutation can transmit different levels of the mutant mtDNA to their children.

The clinical variability of mitochondrial DNA (mtDNA) disease caused by pathogenic mutations in the mitochondrial genome is partly explained by the unique features of mitochondrial genetics (Craven *et al.*2017a). One such feature is the multicopy nature of the mitochondrial genome, meaning that individual human cells can contain from 100 to more than 100,000 copies of mtDNA. In patients who carry a pathogenic mtDNA mutation, this mutation can be present in all copies of the mitochondrial genome (termed homoplasmy) or only a proportion of the genomes (termed heteroplasmy). When heteroplasmy exists, it is the ratio of wild-type to mutant mtDNA that is important and clinical features will only manifest once a critical threshold level has been exceeded. This is complicated further by the presence of a ‘genetic bottleneck’ during development of the female germline, which means that women who carry a pathogenic mtDNA mutation can transmit different levels of mutant mtDNA to their children (Figure 1). This makes genetic counselling difficult as it is not always possible to predict the risk of disease in the child. Moreover, an asymptomatic woman carrying low levels of mutant mtDNA could have a child who is severely affected by mitochondrial disease.

There are a number of reproductive options currently available for women who carry a pathogenic mtDNA mutation and wish to reduce the risk of having a child severely affected by mitochondrial disease. The complexity of mitochondrial genetics, and the fact that not every reproductive option will be suitable for every woman, requires that both mitochondrial and fertility specialists provide advice to couples before an informed decision is made to proceed with a particular option. For those couples wanting a child whose genetic constitution will be inherited from both parents, reproductive options can include becoming pregnant without medical intervention, prenatal testing and preimplantation genetic diagnosis. Importantly, these options will be suitable for women who produce eggs with lower levels of mutant mtDNA but will not reduce the risk of mtDNA disease if a woman produces eggs that contain only high levels of the mutation. Alternate options, which include adoption or egg donation, prevent transmission of mtDNA disease and completely remove the risk of having an affected child. The child will not be genetically related to both parents, however, which may be a consideration for some. More recently, mitochondrial donation has become an additional option that may be suitable for a select group of patients deemed to be at risk of transmitting a serious mtDNA disease and for which PGD will be unsuitable (Gorman *et al.*^2015^). Importantly, no alternative reproductive options exist that allow such women to have a genetically related child, demonstrating a need for mitochondrial donation. At the same time, this does not detract from the other reproductive options that are currently available and aims to provide more reproductive choice. Some have claimed that this assigns too much value to genetic relatedness and assumes that a genetic connection between a mother and her child is essential. A counter argument might be posed that the decision to use a certain reproductive option is a down to personal choice, and for some patients, being genetically related to the child will influence this decision. To support this, recent HFEA figures show that ∼5% of IVF cycles performed in the UK in 2014 used donor eggs.^1^ This implies that most couples opting for fertility treatment choose to pursue a reproductive option that allows them to have a genetically related child when such an option exists; this view is supported by feedback provided from our patient-based focus groups.

**FIGURE 2.**
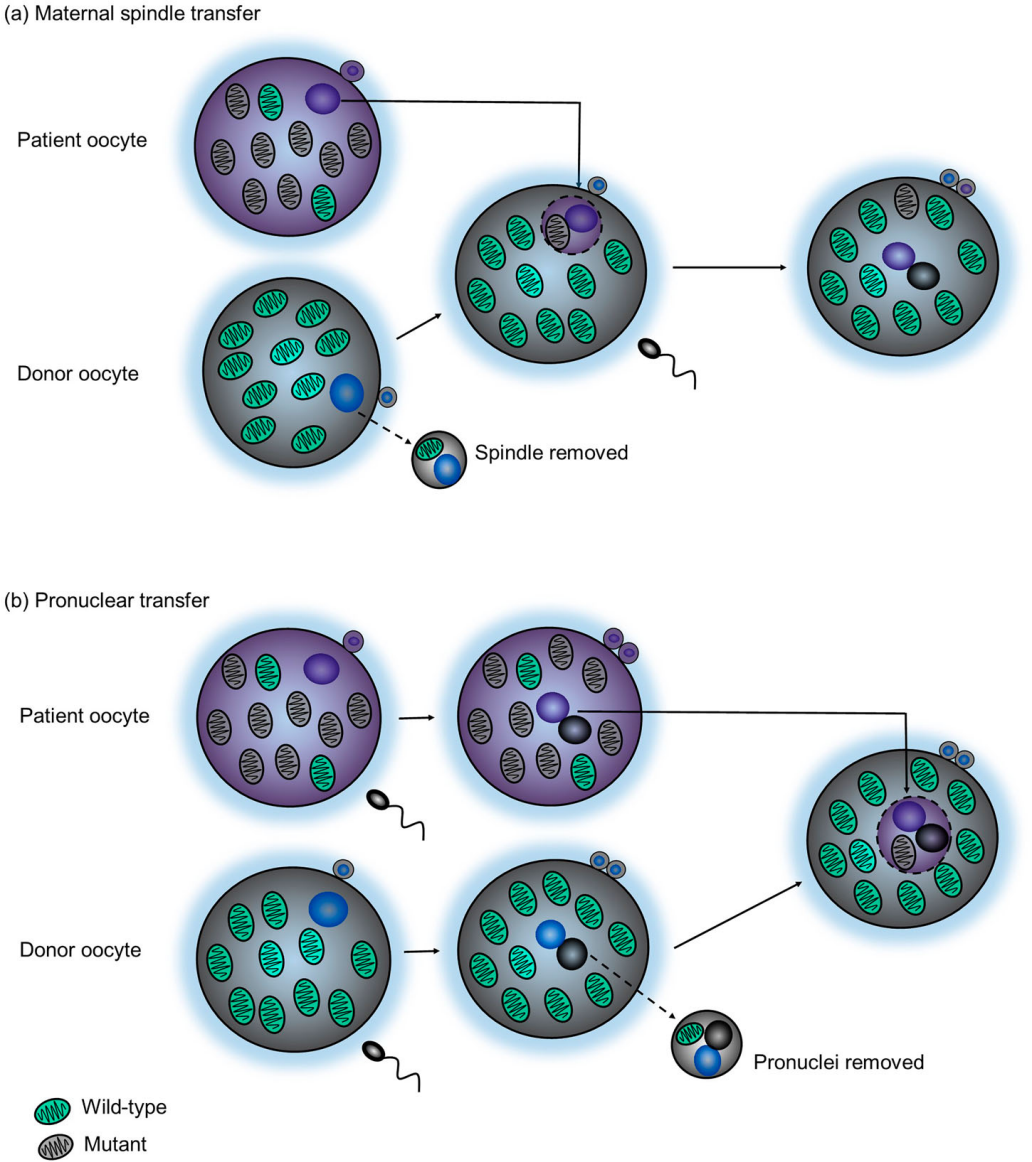
Mitochondrial donation techniques. Mitochondrial donation involves removing the nuclear genome from an oocyte (or zygote) that contains mutant mtDNA and transferring it to a donor oocyte (or zygote) with wild-type mtDNA that has its own nuclear genome removed. The reconstituted oocyte (or zygote) contains the nuclear DNA from the intending parents and the mtDNA from a donor. Mitochondrial donation can be performed between unfertilised human oocytes using a technique called (a) maternal spindle transfer (MST) or between fertilised human zygotes using a technique called (b) pronuclear transfer (PNT).

The process of mitochondrial donation involves removing the nuclear genome from an oocyte (or zygote) that contains mutant mtDNA and transferring it to a donor oocyte (or zygote) with wild-type mtDNA that has its own nuclear genome removed (Figure 2). The reconstituted oocyte (or zygote) contains the nuclear DNA from the intending parents and the mtDNA from a donor, meaning that the resulting child will be genetically related to both parents but will have a much lower risk of developing mtDNA disease. The combination of three people’s DNA, namely the mother and father’s nuclear DNA and the donor’s mtDNA, led to the alternate and now widely used term ‘three parent baby’. This term, coined and often used by the media, is considered inaccurate and has resulted in misconceptions around the technique. At the same time, it is a term that is known by many and has increased awareness of mitochondrial disease, which can ultimately only benefit patients and patient groups.

The development of any novel reproductive technology that involves the manipulation of human embryos will always evoke strong opinions and controversy. This is partly because of the plethora of scientific, ethical and legal issues that surround the clinical use of such techniques. During the policy change that was required to allow the use of mitochondrial donation in the UK, many of these issues were extensively scrutinised by many different people in multiple contexts, resulting in the publication of numerous reports that informed recommendations made to government. In this article, we discuss some of the issues that resonated given our involvement in the development of mitochondrial donation from both a clinical and scientific viewpoint.

## 2. The use of human embryos in research

The experimental techniques required to perform mitochondrial donation, or nuclear transplantation as it was originally known, were first developed in mouse embryos nearly 35 years ago (McGrath and Solter 1983). The original research paper described a successful method for transferring nuclei between fertilised mouse embryos, which allowed good embryo survival and the birth of live animals. At this time, before the first reports of pathogenic mtDNA mutations associated with disease (Holt *et al.*^1988^; Wallace *et al.*^1988^), the potential for the technique to be used to prevent transmission of mtDNA disorders had not been realised. The connection was made a number of years later, and interestingly, one of the first reports that considered the use of nuclear transplantation to ‘cure mitochondrial disease’ was published in a healthcare ethics journal (Rubenstein *et al.*^1995^).

Many research groups performed nuclear transplantation in mouse embryos to address different biological questions (Meirelles and Smith 1997, 1998), but our research group in Newcastle (UK) initially focussed on investigating the potential of the technique to prevent transmission of mtDNA disease. Early studies in mouse embryos went some way to show that the technique could in fact reduce the risk of mtDNA disease (Sato *et al.*^2005^), but disparities between mouse and human embryos meant that the ability to relate these research findings directly to the clinical application of mitochondrial donation was limited. Consequently, the next stage of the research journey required the use of human embryos to determine the safety and efficacy of the technique. This in itself could be considered controversial and raises many ethical questions, the answers to which are complex and influenced by personal views and beliefs.

The need to regulate the use of human embryos in both fertility treatment and scientific research became apparent following the birth of the first *in vitro* fertilisation (IVF) baby in the UK. Not long after this monumental breakthrough, the Warnock Committee was convened to consider the policies and safeguards that should be applied in relation to human fertilisation and embryology, including discussions around whether or not human embryo research should be permitted. Much of this debate focussed on the moral status of the embryo, which is beyond the scope of this article, but is something that remains contentious, continuing to divide opinion across the spectrum of religious and secular beliefs. The inquiry, which was published in the highly esteemed Warnock Report, recommended that research on human embryos should be permitted, but with the restriction that such embryos could only be used for up to 14 days after fertilisation. This rule, which was proposed over 30 years ago, is only now being revisited following recent advances in the *in vitro* culture of human embryos (Deglincerti *et al.*^2016^) and has reignited the ethical debate on embryo research (Cavaliere 2017). The Warnock Report was subsequently used to provide a framework for legislation and formed the basis of the Human Fertilisation and Embryology Act 1990 (HFE Act 1990). This Act, which underwent a major review before publication of the HFE Act 2008, outlines purposes for which human embryos can be used in research. Such research is under strict regulation in the UK by the Human Fertilisation and Embryology Authority (HFEA), who have the power to issue licenses for research projects that involve human embryos. For a license to be granted, the HFEA must be satisfied that the use of human embryos is necessary and that the research fulfils at least one of the purposes outlined in the HFE Act.

The research purposes for which embryo research is permitted include the development of treatments for serious disease or other serious medical conditions, and so our team applied to the HFEA for a license to allow research into mitochondrial donation as a novel IVF treatment to avoid transmission of mtDNA disease. Following an extensive review of the scientific methodology and legal interpretation of the original HFE Act 1990, which highlighted the incredible foresight of the Warnock Committee some years earlier, the Newcastle team were granted the first research licence allowing the use of human embryos to develop the techniques required to perform mitochondrial donation. In the first instance, this was done using abnormally fertilised human embryos often produced during a normal IVF cycle that are unsuitable for clinical use and usually discarded. Importantly, these abnormal embryos can only be used with informed consent from the couple undergoing the IVF treatment following a detailed explanation of the research project.

Early experiments to develop the technique of mitochondrial donation using abnormally fertilised human embryos necessitated many modifications to the methodology used previously in mouse embryos, highlighting the absolute requirement for human embryo research before clinical application can be considered. These ‘proof of principle’ experiments were crucial to demonstrate the potential for mitochondrial donation to prevent transmission of mtDNA disease (Craven *et al.*^2010^) but were restricted by the use of abnormally fertilised embryos. This was mainly because abnormal embryos have a limited capacity for development, which makes it difficult to evaluate both the safety and efficacy of the technique. For this reason, the next stage of the research project required the use of normally fertilised human embryos, which raises more ethical questions surrounding not only the use of these embryos but also the source of the eggs and sperm needed to produce them.

The creation of human embryos for research purposes is allowed in the UK, one of only a few countries that permit this by law. This regulatory position differs throughout the world, with many countries having laws that reflect their own ethical, social and religious frameworks. The creation of human embryos for research was one of the recommendations proposed by the Warnock Committee, who advised that the fertilisation of donated eggs specifically for research purposes should be permissible to avoid confining this research to ‘spare’ embryos (Warnock 1985). There were several reasons why the committee believed this should be permitted, one being that it would increase the validity of the research being undertaken, which would turn out to be crucial for the evaluation of mitochondrial donation before its future clinical application.

The majority of embryos used in research are donated by patients undergoing fertility treatment.^2^ When the research requires the creation of embryos, the eggs can be donated by those undergoing fertility treatment, or so-called ‘egg-sharers’. These are women who donate half of the eggs collected during an IVF cycle for a reduction in their treatment costs (private patients) or an extra IVF cycle if they fail to become pregnant during the IVF cycles offered on the National Health Service (NHS). Ethical concerns have been raised around human eggs being viewed as a commodity and the potential exploitation of women taking part, but a study into the experiences of women who participated in the scheme revealed that they found this not to be the case (Haimes *et al.*^2012^). Furthermore, a review of the outcomes of this scheme revealed that it was successful for both egg sharers and researchers in terms of achieving a pregnancy and allowing research to progress, which is often limited by egg availability (Choudhary *et al.*^2012^). The other source of human eggs for the mitochondrial donation study is non-patient egg donors living in the North East of England, who donate their eggs solely for this research project. This is a very involved process requiring significant commitment and as such, it has been deemed ethically acceptable to provide egg donors with appropriate financial compensation for their time and inconvenience (Hyun 2011). Importantly, this compensation is viewed as insufficient to be considered an inducement. The role of all egg donors in the development of mitochondrial donation as an IVF treatment has been pivotal and their contribution is duly acknowledged because without them, it would not be possible to accurately assess the safety and efficacy of the technique before clinical application.

The continuation of this research with normally fertilised human embryos revealed that the mitochondrial donation technique had to be refined further. This was because the methodology developed with abnormally fertilised human embryos was not well tolerated by normal embryos, with reduced survival following the procedure (Hyslop *et al.*^2016^). This was attributed to accelerated embryo cleavage associated with developmental competence, meaning that the mitochondrial donation technique was being performed too close to the first embryo division. To address this, an alternative approach was developed whereby the procedure was performed much sooner after fertilisation, resulting in improved embryo survival and efficient development to the blastocyst stage, a prerequisite of any IVF treatment being considered for clinical application. In addition, the blastocysts that developed were used to derive embryonic stem cells, which could then be investigated to provide further data regarding the safety of the technique. This analysis revealed that the low level of mtDNA transferred to the donor embryo during the procedure could increase over time in a limited number of stem cell lines, which led the authors to conclude that mitochondrial donation can potentially reduce but not completely eradicate the risk of mtDNA disease and should be considered in combination with prenatal testing. These important issues would not have been identified without the preclinical research, highlighting the importance of using normally fertilised human embryos in such experiments.

## 3. The use of unfertilised or fertilised human oocytes (MST vs PNT)

Mitochondrial donation can be performed at various stages of oocyte development using unfertilised oocytes and techniques such as maternal spindle transfer (MST), polar body transfer (PBT) or germinal vesicle transfer (GVT). Alternatively, it can be performed using fertilised oocytes (zygotes) at the one-cell stage of development and a technique known as pronuclear transfer (PNT) (Craven *et al.*2017b). Both MST and PNT are the most well studied mitochondrial donation techniques to date (Figure 2), with more scientific research to support their clinical use over PBT and GVT. This is reflected in the current UK legislation, namely the Human Fertilisation and Embryology (Mitochondrial Donation) Regulations 2015, which states that only eggs and embryos created following MST or PNT techniques are permitted for clinical use. This ‘permission’ is defined in law and is the result of wide-ranging discussion of the ethical issues around both mitochondrial donation techniques. A consequence of the legal framework for application of these two techniques in the UK is that their use is subject to strict regulatory supervision via the HFEA who issue individual licences on a case by case basis submitted by approved centres.

Although the basic principle of MST and PNT is the same, and the procedures performed only 6-8 hours apart in terms of the timing of preimplantation development, a fundamental difference is that MST is performed using an unfertilised human oocyte that is subsequently fertilised, whilst PNT is performed using two human oocytes that are already fertilised. Consequently, the use of PNT as a clinical treatment could raise more ethical concerns than MST because of the need to create fertilised zygotes that are subsequently manipulated and discarded during the treatment process. These ethical issues are often influenced by personal beliefs and views on the moral status of the embryo, with some having the opinion that MST is more ethically acceptable as it avoids creating an embryo that will ultimately be destroyed. This is perhaps an over simplistic view, however, as it fails to take into account the embryos that have been created and discarded during the crucial research that has taken place to investigate MST as a potential mitochondrial donation technique. Furthermore, although according to UK law a zygote at the pronuclear stage is considered an embryo, this is not a universally accepted definition because the genetic material in both pronuclei has not yet fused to form the nucleus of the embryo. In much the same way, there is an argument that ‘new life’ has not been created until the pronuclei have fused and there has been an exchange of genetic material between parental gametes. With this in mind, the ethical dilemma of PNT may be less convincing and might be considered to have been overplayed. In support of this, the Nuffield Council on Bioethics concluded that it would be ethical to use either MST or PNT in the clinical application of mitochondrial donation and that ongoing research into both techniques was important to establish which technique was likely to be the safest and most effective.^3^ This reflects a view expressed throughout the debates on mitochondrial donation, that the preferred use of MST or PNT should not be dictated solely by ethics, which is heavily influenced by personal views and opinions, but should consider the available safety and efficacy data for each technique.

Given that there is currently no evidence to suggest that MST or PNT is preferable in terms of safety,^4^ it is likely that the decision to use a particular technique will ultimately depend on the expertise of the fertility clinic offering mitochondrial donation. The views of the intending parents may also determine which technique is used, which was reported in the first controversial case of a live birth using MST to prevent transmission of mtDNA disease (Zhang *et al.*^2017^). Here, the authors state that the patient opted for MST over PNT for religious reasons to ‘avoid disrupting a zygote’. However, as previously mentioned, although there may be no ‘disruption’ of a zygote during an individual MST procedure, the research that refined (and continues to improve) this technique has required creation and destruction of numerous human zygotes (Paull *et al.*^2012^; Tachibana *et al.*^2012^; Kang *et al.*^2016^; Yamada *et al.*^2016^). Furthermore, the embryos that were produced in this clinical treatment were biopsied at the blastocyst stage, which ultimately led to the destruction of those embryos that were found to be genetically abnormal. This highlights another ethical conundrum involving other reproductive options such as PGD, which has been used and widely accepted for many years yet leads to the destruction of embryos deemed unsuitable for transfer or cryopreservation. In this regard, mitochondrial donation could raise fewer ethical concerns than PGD if it allowed the creation and subsequent destruction of fewer embryos.^5^

## 4. Safety issues associated with mitochondrial donation

The safety of any new medical intervention, whether it be a new drug or novel treatment, is paramount and must be adequately addressed before it can be used ethically in the clinic. The same is true for mitochondrial donation, emphasising the importance of preclinical research to investigate the safety and effectiveness of any novel reproductive technology (Dondorp and de Wert 2011). This is reflected in the Nuffield Council on Bioethics report, which concluded that it would be ethical for families to use mitochondrial donation techniques provided they are proven to be acceptably safe and effective. This highlights that the ethical use of any treatment is intrinsically linked to its safety, in that it would never be ethical to electively offer a treatment for a non-essential indication (i.e. not life-saving) that was known to be unsafe. Even when such a treatment is life-saving, the use of ‘unsafe’ treatments would be exceptional. The challenge is reaching a consensus on the likely safety issues that may be applicable to the use of mitochondrial donation and then deciding when an appropriate level of safety has been achieved to allow the timely use of the technique in the clinic (Herbert and Turnbull 2018). This arduous task was given to an independent panel of experts, convened by the HFEA, who extensively reviewed the available scientific evidence around the safety and effectiveness of mitochondrial donation on four separate occasions over a period of six years (Greenfield *et al.*^2017^). Their first three reports concluded that there was no evidence to suggest that mitochondrial donation was unsafe for clinical use but proposed additional experiments that would help support this conclusion. The most recent report, published in 2016, went further and declared both MST and PNT sufficiently safe to proceed cautiously with clinical application in the UK under restricted circumstances (http://www.hfea.gov.uk/docs/Fourth_scientific_review_mitochondria_2016.PDF). This is somewhat contentious, however, and there are some who claim we still do not know enough about the safety implications of mitochondrial donation to allow it to be offered in the clinic (Reinhardt *et al.*^2013^).

One such safety issue that became apparent during the preclinical evaluation of mitochondrial donation was the observation that an initial low level of ‘mtDNA carryover’ transferred with the nuclear genome during the mitochondrial donation process could progressively increase in a limited number of embryonic stem cell lines derived from mitochondrial donation embryos (Hyslop *et al.*^2016^; Kang *et al.*^2016^; Yamada *et al.*^2016^). Although this could merely be a feature of stem cell biology, which is possible given the differences between cultured stem cells and post-implantation embryo development, it is obviously a concern as it implies that in a small number of cases, the level of mutated mtDNA could increase during pregnancy and result in a child severely affected by mtDNA disease. This led researchers to conclude that mitochondrial donation has the potential to reduce the risk of mtDNA disease but may not guarantee prevention (Hyslop *et al.*^2016^). For this reason, the expert panel recommended that all patients who become pregnant following MST or PNT are offered prenatal testing. If this test shows that the level of mutated mtDNA has increased, couples may consider terminating the pregnancy. With this in mind, we were concerned about how our patients would react to this research and whether it would change their opinion of the mitochondrial donation technique. To address this, we held a focus group with a small number of patients and their families to inform them of the latest research findings and discuss some of the important outcomes, including the possible risk that mitochondrial donation could result in a child affected by mtDNA disease. The overall feeling was that couples would still consider using the technique despite being aware of the limitations and risks.

This raises two important issues, the first being that it is not possible to know all the potential risks of any new clinical treatment or medical intervention until it is tried for the first time. Preclinical research can allude to potential risks, but the findings may be considered speculative until clinical evaluation. As clinicians and scientists, it is our responsibility to try and understand the available preclinical data and discuss the potential risks with patients before they make an informed decision to proceed. This will involve considering the possible risk to benefit ratio, which is something parents with children affected by mitochondrial disease may have to do on a regular basis. What was poignant from the parliamentary debates around mitochondrial donation was that many parents with children affected by mtDNA disease talked about taking these risks every day, for example, by giving their child many different drugs that often have not been approved for use in paediatric patients, in an attempt to manage the symptoms of their mitochondrial disease. We must give serious consideration and weight to such parental concerns in any assessment of the risk-benefit of offering a technique that prevents the transmission of serious mtDNA disease.

The second issue is the importance of introducing safeguards to protect patients from possible risks until more is known about safety and efficacy of the technique. In this regard, the expert panel made several recommendations, including that consideration is given to ‘haplogroup matching’, a precautionary step that involves selecting a mitochondrial donor with a similar mtDNA sequence to the patient (http://www.hfea.gov.uk/docs/Fourth_scientific_review_mitochondria_2016.PDF). It is noteworthy, however, that research studies which have proposed possible interactions between the nucleus and the mtDNA have been performed using highly inbred animal models that are very different to an outbred human population and as such, the potential risks of nuclear-mitochondrial incompatibility are likely to be low (Eyre-Walker 2017). Therefore, the need to haplogroup match may be unnecessary and could limit use of the technique. A consultation on the clinical use of mitochondrial donation in the USA went one step further and recommended that if clinical trials are permitted, they should consider allowing the transfer of only male embryos following the procedure (http://www.nationalacademies.org/hmd/Reports/2016/Mitochondrial-Replacement-Techniques.aspx). Given the maternal inheritance of mtDNA, this would not constitute germ-line modification and could be considered advantageous until more is known about any potential risks associated with the procedure. This obviously has ethical implications and was rejected in the UK for several reasons. One of these is that sex selection would require additional embryo manipulation beyond that already performed during the mitochondrial donation procedure, which could compromise embryo development and limit the chance of a successful outcome. It would also immediately reduce the number of embryos available for transfer by half (on average), which would reduce the efficiency of the technique and could mean that patients must undergo repeated treatment cycles.

Long term follow-up of any children born will be crucial to confirm the safety and efficacy of mitochondrial donation. Patients and their families are encouraged to take part in this follow-up and, through multiple focus groups, have been very much involved in shaping the overall structure of this care pathway, from antenatal checks during pregnancy right through to a detailed neurodevelopmental assessment of the child at 18 months old. One major way in which patient focus groups influenced the care pathway for children born following mitochondrial donation was in relation to follow-up. It was quickly apparent that while parents were keen to take part in follow-up, they were reluctant to ‘medicalise’ an otherwise healthy child and so wanted to keep hospital visits to a minimum. Taking this into account, the follow-up pathway takes advantage of the multiple health professional encounters that are routine for babies and young children in the UK NHS. Routine and a small amount of additional information will be collected at these time points embedded in the NHS health surveillance for all children and include neonatal hospital discharge assessments, vaccinations and routine health visitor assessments. One significant addition to the follow-up care pathway for children born after mitochondrial donation will be a detailed neurodevelopmental assessment at 18 months old, a time point in development when all children would be expected to be walking and talking. The outcome of this developmental follow-up study will inform the HFEA and the general public on the longer-term safety of the technique. It is anticipated that children will continue to be seen by an expert in paediatric mitochondrial disease, at parental request, until the age of 5 years, providing further long-term data on the neurodevelopmental outcome.

## 5. Engagement and mitochondrial donation

Engaging the public, patients and policy makers in our research has been integral in the development of mitochondrial donation as a clinical treatment. The purpose of this engagement is not only to inform but also to stimulate debate. This was crucial in the lead up to the parliamentary votes that took place on mitochondrial donation, but is something we have continued to do to ensure our research remains patient-focussed.

Before the government would consider changing the law to allow mitochondrial donation in the UK, it was important to gauge public opinion of the technique. To do this, the HFEA were asked to engage with the public and seek views on mitochondrial donation through a public dialogue launched in collaboration with Sciencewise. This extensive public consultation comprised many different project strands, including open consultation meetings and deliberative public workshops. These workshops required that participants attended two events, the first aimed at providing the background knowledge needed to engage in debates around mitochondrial donation and the second focussed on identifying and debating the social and ethical issues around the technique. The overall outcome of the consultation, used by the HFEA to provide advice to government,^6^ was general support for permitting mitochondrial donation in the UK. There were some strong ethical concerns expressed during the consultation but the overall view was that the ethical concerns were outweighed by the arguments in favour of permitting the technique. The consultation was thoroughly evaluated and duly recognised as an exceptional case of public engagement for policy purposes.^7^

We have continued to engage the public with issues surrounding mitochondrial donation. This has involved addressing some of the complex science associated with the technique, which was made more understandable by comparing mitochondria to the ‘batteries of the cell’. This analogy was criticised by some who claimed that it was an oversimplification, but there are others who believe that effective communication to mass audiences should be simple and clear. Support for this is provided by previous public health message over the last 30 years, such as communication around HIV, stroke or smoking, which have all used simple analogies to convey a complex message.

The purpose of our patient engagement has been two-fold. In the first instance, it was important to support the patients and their families who wished to engage with the politicians who would ultimately vote on whether to allow mitochondrial donation in the UK. This provided an opportunity for patients and patient organisations to share their own personal experiences of mitochondrial disease, giving politicians access to knowledge traditionally sourced only from healthcare professionals or scientists. Our engagement and communication strategy considered potential barriers to engagement and to ensure inclusivity, incorporated many different formats including guidance notes, patient newsletters, patient information days and drop-in sessions. The success of this engagement, which also included patients attending parliamentary briefing sessions and speaking openly with politicians about the reality of mitochondrial disease, was apparent during the debates that took place in the Houses of Parliament, with many MPs referring to patients or families with whom they had met. This not only increased awareness of mitochondrial disease, but also empowered patients to actively participate and drive through legislative change. This could partly explain why support for mitochondrial donation was provided by many patients and families even if they would not directly benefit from the technique or consider using it themselves.

Given their involvement in the policy engagement surrounding mitochondrial donation, we have endeavoured to maintain a two-way dialogue with patients and patient organisations that have been front and central in terms of their advocacy for this technique. This allows research updates to be discussed, which ensures that our research focus remains patient-driven, but also provides an opportunity to inform patients about the reproductive options that are currently available. Not all the reproductive options will be suitable to every woman who carries an mtDNA mutation but making patients aware of them from early in reproductive life is something we strive to do. There are benefits and drawbacks to all the reproductive options and intending parents must ultimately decide, with advice from specialists, which one is right for them. It is vital that this decision is well-informed and for this reason, we have set up the Mitochondrial Reproductive Advice Clinic as part of the innovative clinical service we offer in Newcastle. This clinic has a dual purpose, the first being to discuss the available reproductive options with patients and the second to assess fitness for pregnancy, which is obviously important when the intending mother could be affected by mtDNA disease. The information provided in the clinic aims to convey complex mitochondrial genetics in a simple and easy to understand way and there are several formats that have been employed to do this, including the production of a short animation which clearly explains all of the reproductive options available to couples. We have also put together ‘information packs’ containing detailed, though carefully explained, information on the different reproductive options and what each option entails. We feel this is important in patients giving informed consent and to some extent allays claims that both the public debates and media coverage of mitochondrial donation presented this as a ‘straightforward’ solution to help women with mtDNA mutations have a healthy child (Herbrand 2017). The patient information we provide includes a comprehensive overview of each reproductive option with a detailed description of the process required for any IVF-based treatment, with opportunities to discuss further in the mitoART clinic for couples who wish to consider an IVF-based option. This includes information on managing expectation; it is important to inform couples that any IVF treatment can fail at any stage and there are no guarantees of a pregnancy. Consequently, it may be necessary for couples to undergo several cycles of IVF before a pregnancy is achieved. The information we provide has been reviewed by patients and patient organisations to ensure it is both clear and relevant to the people who will access it and subsequently adapted based on their feedback.

## 6. Conclusion

It has been proposed by some that the change in law to allow mitochondrial donation in the UK was rushed and happened before the all scientific and ethical issues around the technique had been fully explored. There are many who would refute this claim by drawing attention to the fact that the issues have been discussed and debated by many separate groups for nearly 18 years (Craven *et al.*^2016^). Furthermore, although the Mitochondrial Donation Regulations came into force in March 2015, the technique is yet to be applied in the UK. This demonstrates the importance placed on responsible innovation and governance of mitochondrial donation, which should be applied to any other reproductive technologies that become available in the future.

The use of mitochondrial donation to prevent the transmission of mtDNA disease is allowed in the UK, the only country to have legislated in this area (Herbert and Turnbull 2017). In reaching a decision to permit this technique, the scientific, ethical and legal issues have been deliberated and importantly, continue to be deliberated to this day. A conclusion reached by many is that it is ethical to support and promote the use of mitochondrial donation but with the view that it is used cautiously and under strict regulation (Bredenoord and Appleby 2017). It must also be noted that mitochondrial donation is only legal in the UK if the purpose is to avoid serious mtDNA disease, with questions remaining around the ethical use of the technique for alternative clinical applications, such as to help with infertility.

## Footnotes

1 https://www.hfea.gov.uk/about-us/news-and-press-releases/2016-news-and-press-releases/new-report-shows-ivf-cycles-are-on-the-rise-but-few-involve-frozen-eggs/.

2 http://hfeaarchive.uksouth.cloudapp.azure.com/www.hfea.gov.uk/hfea/rss/161.html.

3 http://nuffieldbioethics.org/wpcontent/uploads/2014/06/Novel_techniques_for_the_prevention_of_mitochondrial_DNA_disorders_compressed.pdf.

4 http://www.hfea.gov.uk/docs/Fourth_scientific_review_mitochondria_2016.PDF.

5 http://nuffieldbioethics.org/wpcontent/uploads/2014/06/Novel_techniques_for_the_prevention_of_mitochondrial_DNA_disorders_compressed.pdf.

6 http://hfeaarchive.uksouth.cloudapp.azure.com/www.hfea.gov.uk/docs/Mitochondria_replacement_consultation_-_advice_for_Government.pdf.

7 http://webarchive.nationalarchives.gov.uk/20170110134326/, http://www.sciencewise-erc.org.uk/cms/assets/Uploads/Mitochondria-evaluation-FINAL-2013.pdf.
